# Comparison of the *In Vivo* Distribution of Four Different Annexin A5 Adducts in Rhesus Monkeys

**DOI:** 10.1155/2011/405840

**Published:** 2011-05-04

**Authors:** Paul McQuade, Marie-Jose Belanger, Xiangjun Meng, Ilonka Guenther, Stephen Krause, Dinko Gonzalez Trotter, Chris Reutelingsperger, Eric Hostetler, Michael Klimas, Huseyin Mehmet, Jacquelynn Cook

**Affiliations:** ^1^Imaging Research, Merck Research Laboratories, West Point, PA 19486, USA; ^2^Department of Biochemistry, University Maastricht, 6200 NL Maastricht, The Netherlands; ^3^Diabetes Exploratory Biomarker, Merck Research Laboratories, West Point, PA 19486, USA

## Abstract

Annexin A5 has been used for the detection of apoptotic cells, due to its ability to bind to phosphatidylserine (PS). Four different labeled Annexin A5 adducts were evaluated in rhesus monkey, with radiolabeling achieved via 1,4,7,10-tetraazacyclododecane-1,4,7,10-tetraacetic acid (DOTA). Of these adducts differing conjugation methods were employed which resulted in nonspecific radiolabeling (**AxA5-I**), or site-specific radiolabeling (**AxA5-II**). A nonbinding variant of Annexin A5 was also evaluated (**AxA5-II_NBV_**), conjugation here was site specific. The fourth adduct examined had both specific and nonspecific conjugation techniques employed (**AxA5-II_mDOTA_**). Blood clearance for each adduct was comparable, while appreciable uptake was observed in kidney, liver, and spleen. Significant differences in uptake of **AxA5-I** and **AxA5-II** were observed, as well as between **AxA5-II** and **AxA5-II_NBV_**. No difference between **AxA5-II** and **AxA5-II_mDOTA_** was observed, suggesting that conjugating DOTA nonspecifically did not affect the *in vivo* biodistribution of Annexin A5.

## 1. Introduction

Apoptotic cells undergo a structural change in which phosphatidylserine (PS), a phospholipid normally present on the inner leaflet of the cell membrane, becomes externalized and available to bind the highly PS selective protein Annexin A5 [1]. The externalization of PS occurs early in the apoptosis process, therefore Annexin A5 can be used to identify apoptotic cells earlier than other methods [2]. As a consequence many studies have been published on the use of Annexin A5 in noninvasive imaging for the *in vivo* detection of apoptosis utilizing radionuclides such as ^99m^Tc, ^18^F, and ^124^I [3–5]. Most of the work to date has focused on labeling Annexin-V with ^99m^Tc, with hydrazinonicotinamide (HYNIC) being the most common bifunctional chelator (BFC) used to tether ^99m^Tc to Annexin A5 [6, 7]. ^99m^Tc-labeled Annexin A5 has also been used clinically to monitor increases in apoptosis in tumors after treatment [8, 9]. However it should be noted that in tumors, Annexin A5 has been shown to detect not only cells undergoing apoptosis but also necrosis as well as other forms of cell death mechanisms [10, 11].

For protein radiolabeling, BFCs such as HYNIC are typically conjugated to free amines such as lysine residues. In the case of Annexin A5, the presence of multiple lysine residues makes placement of a BFC at a specific location impossible. In theory all of the lysine residues in Annexin A5 could have a BFC attached, however in practice this is not the case as the overall number of BFCs can be controlled by adjusting the stoichiometry of the conjugation reaction. This is important as overconjugation of BFCs can have a detrimental effect on ligand binding [12–14]. Previously, in an attempt to facilitate a more reproducible and selective conjugation of Annexin A5, a single cysteine residue was incorporated using site-specific mutagenesis allowing for site-specific conjugation [6]. This additional cysteine residue was attached at the *N*-terminus, which is well removed from the PS binding region and therefore should have no detrimental effect on PS-Annexin A5 binding [15]. 

The ability of this *N*-terminally modified Annexin A5 to detect PS *in vivo* has been established in animal models in which apoptosis was chemically induced. In one study, both wild-type and *N*-terminally modified Annexin A5 adducts were compared in mice in which hepatic apoptosis was induced by the anti-Fas monoclonal antibody (anti-Fas mAb) [6]. ^99m^Tc was used to radiolabel both Annexin A5 adducts, with both adducts showing comparable increases in liver uptake in mice pretreated with the anti-Fas mAb uptake as compared to control animals. TUNEL staining confirmed increased level of apoptosis in liver after anti-Fas mAb treatment. In a separate study [16], cycloheximide treatment in rats resulted in elevated uptake of ^99m^Tc labeled *N*-terminal modified Annexin A5 in liver and spleen over controls. As in the previous study, TUNEL staining confirmed increased level of apoptosis in liver after cycloheximide treatment. These results demonstrate that the introduction of a cysteine residue to wild-type Annexin A5 does not inhibit its ability to bind to PS and that *N*-terminally modified Annexin A5 has potential for the *in vivo* detection of apoptotic cells.

**Figure 1:**
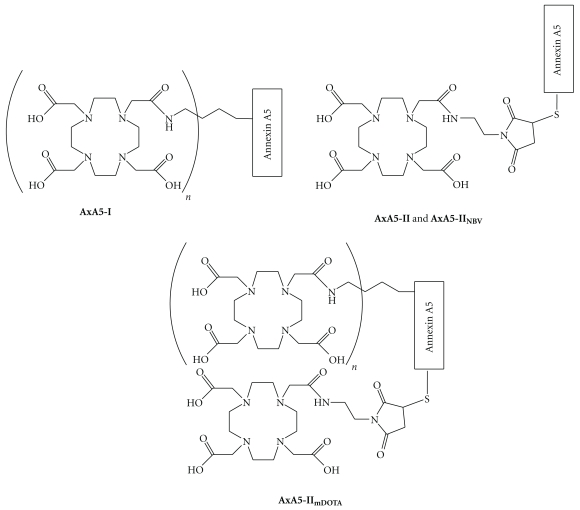
Schematic representation showing conjugation methods employed to tether DOTA non-specifically to multiple (*n* > 1) free amines present in Annexin A5 (**AxA5-I**) or site specifically to the single cysteine residue present on the *N*-terminally modified Annexin A5 (**AxA5-II** and **AxA5-II_NBV_**). For **AxA5-II_mDOTA_** both conjugation methods were employed.

In this report we describe *in vivo* PET imaging studies carried out with four different Annexin A5 adducts in rhesus monkeys. These adducts were radiolabeled with the positron emitting isotope ^64^Cu (*t*_1/2_ = 12.7 h, *β* ^+^ = 17.6%, *E*_avg_ = 278 keV) via the BFC 1,4,7,10-tetraazacyclododecane-1,4,7,10-tetraacetic acid (DOTA). DOTA is one of the most commonly used BFCs used to label biomolecules with ^64^Cu, with imaging experiments being conducted up to 3 days after administration of the radiolabeled biomolecule [17]. In this study, DOTA was conjugated to multiple free amine sites present in wild-type Annexin A5 (**AxA5-I**) or to the single cysteine residue present on the *N*-terminally modified Annexin A5 (**AxA5-II**). Both conjugation methods employed have significant precedence and are well reported in the literature. The primary goal of the study was to compare the biodistribution of **AxA5-II** versus **AxA5-I** to see if there were any factors that would preclude the clinical use of **AxA5-II**. Rhesus monkeys were chosen as the test species, as the pharmacokinetic (PK) parameters in nonhuman primates are more closely related to human PK than other preclinical species [18–20]. Additionally, a nonbinding variant of Annexin A5 (**AxA5-II_NBV_**) was radiolabeled and evaluated to provide insight into potential specific binding of **AxA5-II**. **AxA5-II_NBV_** was similar to **AxA5-II** in that a single cysteine residue incorporated at the *N*-terminus, facilitating site-specific DOTA conjugation. The difference between these Annexin A5 variants was that **AxA5-II_NBV_** contained a single point mutation in each of the 4 domains of Annexin A5 essential for calcium binding. This lack of calcium binding precludes the ability of Annexin A5 to bind to phosphatidylserine, with circular dichroism analysis showing that these mutations did not significantly alter the folding of Annexin A5. Finally, to monitor the effect the conjugation method employed had on the biodistribution and clearance of Annexin A5, a fourth Annexin A5 adduct was evaluated, **AxA5-II_mDOTA_**. For **AxA5-II_mDOTA_** the *N*-terminal functionalized Annexin A5 had DOTA conjugated both non-specifically and on the terminal cysteine. A schematic representation showing the differing conjugation methods employed to tether DOTA to Annexin A5 is shown in Figure 1.

## 2. Materials and Methods

### 2.1. General

All chemicals, unless otherwise stated, were purchased from Sigma-Aldrich Chemical Co. (St. Louis, MO). Water was distilled and then deionized (18 MΩ/cm^2^) by passing through a Milli-Q water filtration system (Millipore Corp., Milford, MA). ^64^Cu was purchased from either Washington University School of Medicine (St. Louis, Missouri) or MDS Nordion (Ottawa, Canada). Wild-type, *N*-terminal cysteine functionalized and *N*-terminal cysteine functionalized scrambled Annexin A5 were obtained from MosaMedix BV, The Netherlands. 1,4,7,10-tetraazacyclododecane-1,4,7,10-tetraacetic acid mono(*N*-hydroxysuccinimidyl ester) and 1,4,7,10-Tetraazacyclododecane-1,4,7-tris-acetic acid-10 maleimidoethylacetamide were obtained from Macrocyclics (Dallas, TX).

### 2.2. Chemistry

#### 2.2.1. DOTA Conjugation of Annexin A5 Analogs

**AxA5-I**

Wild-type Annexin A5 (1 mg, 27.8 nmol) and 1,4,7,10-tetraazacyclododecane-1,4,7,10-tetraacetic acid mono(*N*-hydroxysuccinimidyl ester) (0.23 mg, 0.28 *μ*M) were stirred together for 18 hr at 4°C in Na_2_HPO_4_ (0.1 M, pH 8.5). The reaction mixture was transferred to a Centricon YM-10 Centrifugal Filter (Millipore Corp., Milford, MA) where ammonium citrate (1.5 mL, 0.1 M, pH 5.5) was added and then centrifuged for 20 min. The supernatant was discarded and an additional aliquot of ammonium citrate (4 mL) was added. It was again centrifuged for 20 min and the supernatant was discarded. This process was repeated 3 times. Purified **AxA5-I** was collected in ammonium citrate (200 *μ*L), the concentration determined by UV absorbance and the **AxA5-I** solution, was stored for up to 5 months at 4°C.

**AxA5-II and AxA5-I I_NBV_**

Either* N*-terminal cysteine-modified Annexin A5 or the *N*-terminal cysteine-modified scrambled Annexin A5 variant (3.6 mg, 50 nmol) was dissolved in HEPES buffer (500 *μ*L, 25 mM, pH 7.2). Dithiothreitol (1.57 mg, 10.2 nmol) was added and the resulting solution diluted to 1 mL with HEPES buffer. The reaction mixture was incubated at 37°C for 60 minutes and transferred to a Centricon YM-10 Centrifugal Filter where an additional aliquot of HEPES buffer was added (3.0 mL). The resulting solution was centrifuged for 20 min, and the supernatant discarded. Additional HEPES buffer (4 mL) was added and the centrifugation process repeated. The reduced Annexin A5 was collected in HEPES buffer (200 *μ*L) and 1,4,7,10-tetraazacyclododecane-1,4,7-tris-acetic acid-10 maleimidoethylacetamide (0.1 mg, 100 nmol) was added. The solution was diluted to 1 mL by the addition of HEPES buffer. The resulting reaction mixture was incubated at 37°C with gentle mixing for 18 h. The reaction mixture was transferred to a Centricon YM-10 Centrifugal Filter where ammonium citrate (3.0 mL, 0.1 M, pH 5.5) was added. After centrifugation for 20 min, the supernatant was discarded, and an additional aliquot of ammonium citrate (4 mL) was added. It was again centrifuged for 20 min and the supernatant discarded. This process was repeated three times. Purified **AxA5-II **or **AxA5-II_NBV_** were collected in ammonium citrate (200 *μ*L), the concentrations were determined by UV absorbance, and the solutions were stored for up to 5 months at 4°C.

**AxA5-I I_mDOTA_**

DOTA was conjugated to the single cysteine residue present on the *N*-terminal cysteine functionalized Annexin A5 via 1,4,7,10-tetraazacyclododecane-1,4,7-tris-acetic acid-10 maleimidoethylacetamide using identical reaction conditions to those described for **AxA5-II** and **AxA5-II_NBV_**. After initial purification, DOTA was conjugated in a nonsite-specific fashion using 1,4,7,10-tetraazacyclododecane-1,4,7,10-tetraacetic acid mono(*N*-hydroxysuccinimidyl ester) in conditions identical to those described for **AxA5-I**. After final purification, **AxA5-II_mDOTA_** was collected in ammonium citrate (200 *μ*L), the concentration determined by UV absorbance and the **AxA5-II_mDOTA_** solution stored for up to 5 months at 4°C.

### 2.3. Radiochemistry

^64^Cu radiolabeling experiments with all DOTA functionalized Annexin A5 adducts were performed in an identical fashion as follows: to the DOTA-conjugated Annexin A5 adduct was added ^64^CuCl_2_ in 0.1 M HCl and the mixture diluted with ammonium citrate (0.1 M, pH 5.5) to a volume of 100 *μ*L. The reaction mixture was incubated at 37°C for 30 min, allowed to cool for 5 min before diethylenetriaminepentaacetic acid (DTPA, 3 *μ*L, 10 mM) was added. The ^64^Cu-labeled Annexin A5 was purified via a Bio-Spin 30 column (Bio-Rad, Hercules CA) that had been pretreated with 3 × 1 mL of phosphate buffered saline (PBS). Radiochemical purity was determined by injection on an analytical Waters 2795 HPLC system (Milford, MA) equipped with a Waters 996 UV detector and **β**-RAM Model 4 Radio-HPLC detector (IN/US Systems, Brandon FL) using a TSKgel G3000SW_XL_ size exclusion column and PBS as the mobile phase. All ^64^Cu labeled Annexin A5 adducts eluted with a retention time of 8.9 min. This retention time matches that observed for all nonradiolabeled Annexin A5 adducts as well as wild-type Annexin A5.

### 2.4. Red Blood Cell Calcium Titration Assay

The relative binding of ^64^Cu-labeled **AxA5-I**, **AxA5-II** or **AxA5-II_NBV_** to phosphatidylserine was determined by binding to RBC at various Ca^2+^ concentrations [21]. A fixed concentration of ^64^Cu-labeled **AxA5-I**, **AxA5-II** or **AxA5-II_NBV_** along with a fixed number of preserved red blood cells (Beckman Coulter “4C ES normal” Hialeah, FL) was titrated against increasing concentrations of CaCl_2_ (0–6 mM), with each concentration tested in duplicate. After incubation for 8 min at room temperature, the cells were pelleted from the media by centrifugation and the supernatant was removed. The pellets were then resuspended in assay buffer containing CaCl_2_ at the same concentrations as the original incubation buffer, centrifuged a second time and the supernatant was discarded. The level of ^64^Cu-labeled Annexin A5 bound to each red blood cell pellet was determined by measuring the counts per minute (CPM) using a gamma counter (1480 Wizard 3^”^, PerkinElmer, Waltham MA).

### 2.5. PET Imaging Studies

PET studies were conducted in healthy adult male rhesus monkeys (*n* = 3, weight 9.27 ± 0.57 kg), with the same 3 animals used for each annexin adduct. All procedures were conducted in accordance with the guidelines of the Institutional Animal Care and Use Committee of Merck (West Point, PA) and guidelines for the care and use of mammals in neuroscience and behavioral research (National Research Council 2003). Animals were housed in temperature- and humidity-controlled rooms in fully AAALAC (Association for Assessment and Accreditation of Laboratory Animal Care) accredited facilities and fed a commercially prepared high protein monkey diet (Lab Diet no. 5045, PMI Nutrition International Inc., Brentwood, MO); water was offered ad libitum. Fresh fruits and vegetables were provided daily and animal housing rooms were maintained on a twelve hour light/dark cycle.

Animals were initially sedated with Ketamine Hydrochloride (10 mg/kg IM) anesthesia and maintained with IV Propofol anesthesia (5 mg/kg for induction and 0.4 mg/kg/min throughout the scanning procedure). Following the initial induction with Propofol, the animal was intubated and ventilated with medical grade compressed air at ~10 cc/breath/kg and 20 respirations per minute. Monkeys were instrumented with a temperature probe, a pulse oximeter and an end tidal CO_2_ monitor. End tidal CO_2_ was maintained at 40 ± 2 mm Hg, with body temperature maintained between 98.5–100°F using K-module heating pads (Harvard Apparatus, Holliston, MA).

Following IV administration of the radiotracer a whole body dynamic scan (180 min) was performed encompassing five fields of view (24 static frames). 24 h after tracer injection a single whole body image was acquired. The initial **AxA5-II** 3 h dynamic scan was acquired using a Siemens HR+ PET scanner (Siemens Medical Solution, Hoffman Estates, IL). All subsequent PET scans were performed using a GE Discovery ST PET/CT (GE Healthcare, Waukesha, WI, USA). Both PET scanners were cross-calibrated with the dose calibrator using an ^18^F cylinder according to the manufacturer's standard operating procedures. 

For whole body images acquired using the GE PET/CT, each image consisted of 5 bed positions covering 72.9 cm axially (voxel size: 2.4 mm × 2.4 mm × 3.3 mm). Images were acquired in 2D mode with random correction from singles and reconstructed with Ordered-Subsets Expectation-Maximization (OS-EM) using 2 iterations and 30 subsets. CT-based attenuation and scatter correction was carried out as implemented by the camera manufacturer. A Gaussian filter (FWHM = 3 mm) was applied to the reconstructed PET image. Before the PET acquisitions, a noncontrast CT (60 mA, 120 kVp) was acquired for PET attenuation correction. After completion of the whole body scan, a contrast CT (200 mA, 120 kVp) using 2.5 mL/kg Omnipaque 300 was performed to assist with organ identification. 

Whole body images acquired with the Siemens HR+ PET scanner consisted of 5 bed positions covering 69.4 cm axially (voxel size: 2.6 mm × 2.6 mm × 2.6 mm). Images were acquired in 2D mode and reconstructed using filtered back-projection with scatter and ^68^Ge 10 min/bed transmission-based attenuation correction as implemented by the camera manufacturer. 

For the 3 h dynamic scan, regions of interest were drawn for the entire left kidney and liver using the summed PET image, while a 5 mm radius sphere was placed inside the left ventricle of the heart using the initial PET image. For the 24 h scans, regions of interest were drawn for left kidney, liver, and spleen using the PET image. All regions were decay corrected to time of injection and expressed as standardized uptake values (SUV) which was calculated for each region of interest at time point t as the ratio of decay-corrected tissue radioactivity concentration (MBq/mL) at time *t*, *c*(*t*), and *injected dose* (MBq) at the time of injection (*t* = 0) divided by *body weight* (g).

### 2.6. Statistical Methods

Sigma-Stat v3.1 was used for statistical testing. One-way analysis of variance (ANOVA) tests were used to compare results among groups, with all after hoc pairwise comparisons done using the Fisher LSD method. Statistical significance was set at *P* < .05. For groups in which no statistical differences were observed, the statistical analysis should be treated with caution due to the low number of animals (*n* = 3) used.

## 3. Results

### 3.1. Radiolabeling

The DOTA conjugated Annexin A5 adducts were labeled by incubating them with ^64^Cu at 37°C for 30 min. Upon purification by a Bio-Spin 30 column, the ^64^Cu-labeled Annexin A5 adducts were obtained with high radiochemical purity and specific activity (Table 1).

### 3.2. Red Blood Cell Calcium Titration Assay

**Figure 2:**
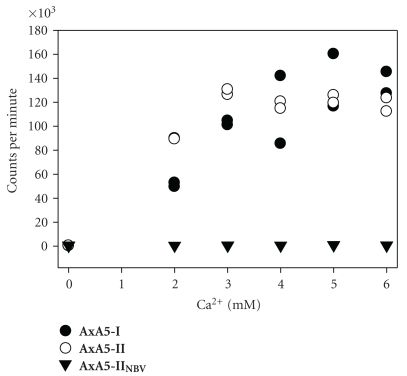
Binding results of ^64^Cu-labeled **AxA5-I**, **AxA5-II**, and **AxA5-II_NBV_** to preserved RBCs with exposed phosphatidylserine at increasing Ca^2+^ concentrations. Measurements were performed in duplicate for each Ca^2+^ concentration.

The RBC titration binding assay showed that ^64^Cu-labeled **AxA5-I**, **AxA5-II** and **AxA5-II_NBV_** had negligible binding to RBCs in the absence of Ca^2+^. For both **AxA5-I** and **AxA5-II**, there was a corresponding increase in RBC binding as the Ca^2+^ concentration increased, with maximum binding reached seen at a Ca^2+^ concentration above 3 mM. For **AxA5-II_NBV_** no binding to the RBCs was observed regardless of the Ca^2+^ concentration (Figure 2). 

### 3.3. PET Imaging Studies

**Figure 3:**
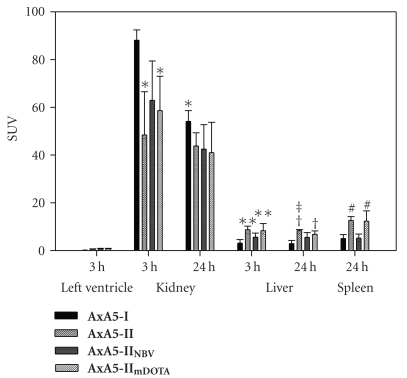
SUVs as determined by drawing regions of interest around target tissue and expressed as Mean ± SD (*n* = 3) of target organs at end of 3 h dynamic scan and/or 24 hr after injection of ^64^Cu-labeled Annexin A5 adducts. *P < .05 versus 3 h **AxA5-I** Kidney, **P < .05 versus 3 h **AxA5-I** Liver, ^†^ *P* < .01 versus 24 h **AxA5-I** Liver, ^‡^ *P* < .05 versus 24 h **AxA5-II_NBV_** Liver, ^#^ *P* < .01 versus 24 h **AxA5-I** and **AxA5-II_NBV_** Spleen.

**Figure 4:**
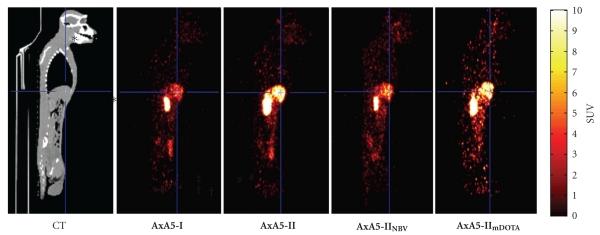
Summed whole body PET images from 24 h of ^64^Cu labeled **AxA5-I**, **AxA5-II**, **AxA5-II_NBV_**, and **AxA5-II_mDOTA_** showing liver (crosshairs) and right kidney. PET images obtained with the same rhesus monkey and are shown along with a whole body contrast CT scan obtained after the **AxA5-I** scan.

For each ^64^Cu-labeled Annexin A5 adduct, two PET scanning sessions were conducted and the results are shown in Figure 3. Summed whole body PET images of each adduct at 24 h in the sagittal plane are shown in Figure 4. 

The first, a 3 h dynamic scan, began immediately following administration of the radiotracer. Clearance from the blood of all four adducts was comparable as judged by the SUV levels in the left ventricle, with levels <1 SUV seen for all four adducts by 3 h. Each adduct behaved similarly during this initial dynamic scan, with highest uptake in kidney followed by liver. **AxA5-I** had the highest kidney SUV value; 88.1 ± 4.3 at 3 h, which was significantly higher than both **AxA5-II** (*P* = .01) and **AxA5-II_mDOTA_** (*P* = .04). 

For each Annexin A5 adduct a second PET imaging session was performed 24 h after radiotracer administration. As was the case at 3 h, the kidney was still the organ of highest uptake for all adducts. However, at 24 h only **AxA5-I** showed a significant drop in kidney uptake compared to 3 h, 88.1 ± 4.3 versus 54.1 ± 4.5 (*P* = .003). In terms of liver uptake, each adduct showed no significant reduction at 24 h as compared to 3 h. Similar to what was seen at 3 h, at 24 h liver uptake of both **AxA5-II **and **AxA5-II_mDOTA_**was significantly higher than **AxA5-I**; *P* = .001 and  .009 respectively. Additional, by 24 h, liver uptake of **AxA5-II** was significantly higher than **AxA5-II_NBV_** (*P* = .04). 

At the 24 h time point the spleen could also be readily identified. During the initial 3 h dynamic scan a region of interest for the spleen could not be drawn accurately as there was poor delineation between it and the left kidney. Spleen SUV uptake of **AxA5-II** was significantly higher than both **AxA5-I** (*P* = .008) and **AxA5-II_NBV_** (*P* = .008). Similar to **AxA5-II**, spleen uptake** AxA5-II_mDOTA_** was also significantly higher than both **AxA5-I** (*P* = .009) and **AxA5-II_NBV_** (*P* = .01).

## 4. Discussion

The primary goal of this study was compare the biodistribution of wild-type Annexin A5 radiolabeled in a nonsite-specific fashion (**AxA5-I**) to an Annexin A5 analog amenable to site-specific radiolabeling by the incorporation of a single cysteine residue at the *N*-terminus (**AxA5-II**). This was done to see if any significant differences in the *in vivo* distribution of **AxA5-I** and **AxA5-II** existed, and if these differences would preclude the clinical use of **AxA5-II**. In addition, two other Annexin A5 analogues were examined: **AxA5-II_NBV_** and **AxA5-II_mDOTA_**. **AxA5-II_NBV_** was radiolabeled in an identical fashion as **AxA5-II**; however the amino acid sequence had been scrambled to remove PS affinity. **AxA5-II_NBV_** was evaluated to see if any **AxA5-II** uptake could be attributed to specific PS binding. For **AxA5-II_mDOTA_**, both DOTA conjugation methods were employed to discover if this had any effect on the *in vivo* distribution of Annexin A5. These adducts were evaluated in rhesus monkeys as numerous studies have shown that the pharmacokinetic parameters displayed in nonhuman primates is more predictive of human parameters than is achievable with other more commonly available laboratory species [18–20].

Before *in vivo* imaging work was undertaken, *in vitro* studies based upon the previously reported red blood cell (RBC) calcium titration assay were carried out [21]. Determination of Annexin A5 affinity using this assay is possible since the binding of Annexin A5 to PS is dependent on the available Ca^2+^ concentration [22]. PS expression on the cell surface of RBCs increases with age, therefore, in the presence of Ca^2+^, Annexin A5 will bind to RBCs [23]. For a fixed concentration of Annexin A5 and PS, which is maintained by keeping the number of RBCs constant, there should be a gradual increase in binding of Annexin A5 to RBCs as the Ca^2+^ concentration is increased, with maximum binding being achieved at some Ca^2+^ concentration. **AxA5-I** and **AxA5-II** were examined to see if DOTA conjugation affected their ability to bind to PS, and **AxA5-II_NBV_** was examined as a negative control since no binding would be expected at any Ca^2+^ concentration with this variant. All three Annexin A5 adducts showed very low binding to RBCs in the absence of Ca^2+^. However, as the Ca^2+^ concentration increased, both **AxA5-I** and **AxA5-II** showed a gradual increase in RBC binding with maximum binding at a Ca^2+^ concentration above 3 mM. As expected, for **AxA5-II_NBV_** no change in RBC binding was observed regardless of the Ca^2+^ concentration. 

For the *in vivo* imaging experiments, each ^64^Cu-labeled Annexin A5 adduct was given as a bolus IV injection. Although differences in specific activity between the various Annexin A5 adducts were observed, the overall mass of any Annexin A5 variant administered was between 2–8 nmol. At such low levels, variations in the amount of Annexin A5 administered should have no impact on either its clearance or distribution. A 3 h dynamic scan began immediately after administration of the tracer and a second imaging session was conducted 24 h after radiotracer administration. The data showed that while the *in vivo* distribution of the four adducts in rhesus monkeys followed a similar pattern, some significant differences in organ uptake were observed. After the initial bolus injection of the radiolabeled Annexin A5 adducts, all four adducts showed fast blood clearance as determined by SUV levels in the left ventricle. In each case during this initial 3 h scan, both kidney and liver were found to have moderate to high uptake of radioactivity, with kidney being the organ of highest uptake for all four adducts. This is not surprising since Annexin A5 at 36 kDa is lower than the 60 kDa renal excretion limit [24]. Compounds with a molecular weight greater than 60 kDa are excreted primarily via the liver. This is also similar to previously reported clinical studies with ^99m^Tc radiolabeled Annexin A5, where the kidney was the organ of highest uptake [25–27]. For all ^64^Cu-labeled Annexin A5 adducts examined, kidney uptake increased steadily after IV administration before reaching their peak uptake by 60 min, with levels remaining unchanged by 3 h. Kidney uptake of **AxA5-I** was significantly higher than both **AxA5-II** (*P* = .02) and **AxA5-II_mDOTA_** (*P* = .036) at 3 h. Additionally, **AxA5-I** showed a statistically significant reduction in kidney uptake by 24 h as compared to 3 h (*P* = .003). No such reduction in uptake at 24 h was seen for **AxA5-II**, **AxA5-II_NBV_**, or **AxA5-II_mDOTA_**. An explanation for these differences is unknown at present. However, it has been reported that kidney uptake of Annexin A5 is predominately non-specific in nature [28], and can be diminished through the incorporation of various chelating moieties [29, 30]. It would therefore be a reasonable assumption that the method used to conjugate DOTA to Annexin A5 could have an effect on kidney uptake and clearance, as could the overall number of DOTA conjugates present. This is an important question, as **AxA5-I** has multiple DOTA moieties conjugated to free amines in a nonspecific fashion, where **AxA5-II** has a single DOTA conjugated to the *N*-terminal cysteine incorporated via site-specific mutagenesis. **AxA5-II_mDOTA_** was investigated in an attempt to address this question, as both DOTA conjugation methods were applied: DOTA was conjugated both at the *N*-terminal cysteine similar to **AxA5-II**, and non-specifically to multiple free amines as with **AxA5-I**. Therefore, any differences observed between **AxA5-II** and **AxA5-II_mDOTA_** could be attributed to the additional DOTA moieties present as a result of non-specific conjugation. When the SUV data generated for **AxA5-II** and **AxA5-II_mDOTA_** was analyzed however no difference in kidney uptake or clearance was observed. This data shows that the additional DOTA moieties present in **AxA5-II_mDOTA_** as compared to **AxA5-II** caused no change in the clearance of Annexin A5 and cannot be used to explain the differences seen between **AxA5-I** and **AxA5-II**. These differences must therefore be a result of the additional cysteine residue found in **AxA5-II** or in its DOTA conjugation.

As previously mentioned, during the initial 3 h dynamic scan in addition to the kidney, the liver was the only other organ with appreciable uptake in which accurate SUVs could be generated. Similar to the kidney, by 3 h there were significant differences in uptake between **AxA5-I** and **AxA5-II** (*P* = .01), this was repeated at 24 h (*P* = .001). In addition to the kidney and liver, for all four adducts the spleen was the only other organ which showed appreciable uptake. However, due to high kidney spillover, accurate measurement during the initial 3 h dynamic scan was not possible. Reliable data for the spleen could only be generated at the 24 h time point. As in the liver, uptake of **AxA5-II** in the spleen was significantly higher than **AxA5-I** (*P* = .005), as was **AxA5-II_mDOTA_** (*P* = .009). Importantly, there were no significant differences in liver or spleen uptake between **AxA5-II** and **AxA5-II_mDOTA_**. This correlates to what was seen with both these adducts in the kidney, in that the additional DOTA moieties present in **AxA5-II_mDOTA_** caused no change in the uptake or clearance of Annexin A5.

The differences in kidney, liver, and spleen uptake seen between **AxA5-I** and **AxA5-II** are not without precedent. In a previous reported study in which ^99m^Tc radiolabeled wild-type Annexin A5 and *N*-terminally cysteine modified Annexin A5 were compared in NMRI mice, similar differences in organ uptake were found [6]. The ^99m^Tc-labeled species in this study were similar to **AxA5-I** and **AxA5-II**, the difference being the bifunctional chelator hydrazinonicotinamide (HYNIC) was used to tether ^99m^Tc to Annexin A5. However, the chemistry employed for the HYNIC conjugation was similar to that used here for DOTA conjugation. The cause of the discrepancy seen in the *in vivo* distribution of **AxA5-I** and **AxA5-II** is as yet unknown. But from the comparison between **AxA5-II**, which had DOTA conjugated site specifically and **AxA5-II_mDOTA_** in which DOTA was conjugated both site specifically and non-specifically in a manner identical to the DOTA conjugation found in **AxA5-I**, it is known that the additional DOTA moieties present as a result of non-specific conditions did not cause these differences. Rather, the source of the differences found between **AxA5-I** and **AxA5-II** must be a result of the additional cysteine present in **AxA5-II** or in its DOTA conjugation.

To see if any uptake of **AxA5-II** could be attributed to PS binding, a nonbinding variant of **AxA5-II** (**AxA5-II_NBV_**) was evaluated. **AxA5-II** and **AxA5-II_NBV_** differed only in that a single amino acid residue in each of the 4 domains of Annexin A5 responsible for the binding of calcium had been replaced. This deletion of calcium binding precludes the ability of **AxA5-II_NBV_** to bind to PS, with circular dichroism showing that these point mutations did not alter the folding of **AxA5-II_NBV_** as compared to **AxA5-II**. Therefore, any differences in their *in vivo* uptake could highlight potential specific PS binding. Blood clearance of both **AxA5-II** and **AxA5-II_NBV_** as well as kidney uptake at both 180 min and 24 h was similar. However uptake of **AxA5-II** was significantly higher than **AxA5-II_NBV_** in both liver (*P* = .039) and spleen (*P* = .008) at 24 h. This evidence suggests that elevated uptake of **AxA5-II** as compared to **AxA5-II_NBV_** in both organs could be driven by specific binding to PS, however further work would be required to confirm this. Analogous findings though have also been reported previously [28]. In this case, similar reductions in spleen and liver uptake of Annexin A5 were caused by reducing its PS affinity by modifying one or more of the four binding domains.

## 5. Conclusion

From *in vitro* analysis and consistent to what was previously reported, incorporation of a cysteine residue into the *N*-terminus of Annexin A5 and subsequent selective DOTA conjugation did not impair PS binding as compared to wild-type Annexin A5, in which DOTA was conjugated in a nonspecific fashion. An *in vivo* evaluation in rhesus monkeys showed that while significant differences in the biodistribution of **AxA5-I** versus **AxA5-II** were found, these differences do not suggest any liability for the clinical use of **AxA5-II** for the detection of apoptosis *in vivo*. However, the cause of the differences in uptake observed between **AxA5-I** and **AxA5-II** is not clear, though it must be a result of the additional cysteine present in **AxA5-II** or in its DOTA conjugation. This assumption can be made as when **AxA5-II **(specific DOTA conjugation) and **AxA5-II_mDOTA_** (specific and non-specific DOTA conjugation) were compared, no significant differences in their biodistribution in rhesus monkey were found. This comparison indicates that conjugating DOTA non-specifically did not cause the differences in tissue uptake and clearance seen between **AxA5-I** and **AxA5-II**. Finally, the higher uptake of **AxA5-II** in the spleen and liver at 24 h as compared to **AxA5-II_NBV_** could suggest specific binding of **AxA5-II **to PS in these organs, though further work would have to be undertaken to confirm this hypothesis.

## Figures and Tables

**Table 1:** Radiochemical purity and specific activity of annexin A5 adducts, Mean ± SD (*n* = 3).

	**AxA5-I**	**AxA5-II**	**AxA5-II_NBV_**	**AxA5-II_mDOTA_**
% Radiochemical Purity	98.2 ± 3.2	96.5 ± 3.6	98.9 ± 0.1	96.0 ± 4.4
Specific activity (GBq/*μ*mol)	43.0 ± 14.7	27.2 ± 15.7	24.1 ± 2.3	16.4 ± 20.5
Activity injected (MBq)	88.8 ± 33.3	99.9 ± 7.4	81.4 ± 22.2	48.1 ± 40.7
